# Sequencing and Analysis of the Sex Determination Region of *Populus trichocarpa*

**DOI:** 10.3390/genes11080843

**Published:** 2020-07-24

**Authors:** Ran Zhou, David Macaya-Sanz, Jeremy Schmutz, Jerry W. Jenkins, Gerald A. Tuskan, Stephen P. DiFazio

**Affiliations:** 1 Department of Biology, West Virginia University, Morgantown, WV 26506-6057, USA; razhou@mix.wvu.edu (R.Z.); david.macayasanz@mail.wvu.edu (D.M.-S.); 2 HudsonAlpha Institute of Biotechnology, 601 Genome Way Northwest, Huntsville, AL 35806, USA; jschmutz@hudsonalpha.org (J.S.); jjenkins@hudsonalpha.org (J.W.J.); 3 Department of Energy Joint Genome Institute, 2800 Mitchell Drive, Walnut Creek, CA 94598, USA; tuskanga@ornl.gov; 4 Center for Bioenergy Innovation, Biosciences Division, Oak Ridge National Lab, Oak Ridge, TN 37830, USA

**Keywords:** sex, *Populus*, genome, inverted repeats, cytokinin response regulator

## Abstract

The ages and sizes of a sex-determination region (SDR) are difficult to determine in non-model species. Due to the lack of recombination and enrichment of repetitive elements in SDRs, the quality of assembly with short sequencing reads is universally low. Unique features present in the SDRs help provide clues about how SDRs are established and how they evolve in the absence of recombination. Several *Populus* species have been reported with a male heterogametic configuration of sex (XX/XY system) mapped on chromosome 19, but the exact location of the SDR has been inconsistent among species, and thus far, none of these SDRs has been fully assembled in a genomic context. Here we identify the Y-SDR from a Y-linked contig directly from a long-read PacBio assembly of a *Populus trichocarpa* male individual. We also identified homologous gene sequences in the SDR of *P. trichocarpa* and the SDR of the W chromosome in *Salix purpurea*. We show that inverted repeats (IRs) found in the Y-SDR and the W-SDR are lineage-specific. We hypothesize that, although the two IRs are derived from the same orthologous gene within each species, they likely have independent evolutionary histories. Furthermore, the truncated inverted repeats in *P. trichocarpa* may code for small RNAs that target the homologous gene for RNA-directed DNA methylation. These findings support the hypothesis that diverse sex-determining systems may be achieved through similar evolutionary pathways, thereby providing a possible mechanism to explain the lability of sex-determination systems in plants in general.

## 1. Introduction

The evolution of sex is a fundamental yet complex mystery to biologists. Sexual selection has long been recognized as a potent force in evolution [1], sometimes even superceding the effects of other forms of natural selection to drive traits to extreme values, resulting in striking sexual dimorphism [2]. In recent years, it has become increasingly clear that there are a wide variety of genetic mechanisms of sex determination in plants [3]. Unlike gonochorous animals, angiosperm plants are largely co-sexual, meaning that each individual has both sex functions. Some co-sexual species have hermaphroditic flowers, and some are monoecious, where pistils and stamens are present on different flowers within the same individual. Dioecious species represent only about 5% of plants, but these are spread across many angiosperm taxa, suggesting numerous instances of independent evolution [3,4]. Another difference between animals and plants is that a range of reproduction modes can be found in just one genus, e.g., the genus *Silene*, which contains hermaphroditism, dioecy, and several intermediate modes as well [5].

Sex chromosomes are generally considered to have evolved from a pair of autosomes with arrested recombination around the sex-determining loci [6]. The cessation of recombination, along with chromosomal rearrangements, contributes to the further divergence of the proto sex chromosomes, which eventually leads to fully established sex chromosomes [6,7]. Two main sex determination systems are commonly seen in animals and plants. One is female heterogametic, or ZW/ZZ, where females carry a pair of alternate sex chromosomes; the other is male heterogamety, or XX/XY systems, where males carry the alternate sex chromosomes. With the advent of long read sequencing technologies and improved genome assemblies, it became possible to analyze characteristic features of plant sex chromosomes, thereby gaining insights into their functions and evolution. Many sex chromosomes in plants are homomorphic, in contrast to the preponderance of strongly heteromorphic sex chromosomes in animals. Homomorphism could indicate young ages or slow evolution of sex chromosomes, and this remains an open question that can be resolved in part by assembling and aging more independently evolved homomorphic sex chromosomes. Studies based on model animal species can lead to a false impression of the conservation of sex determination mechanisms [8]. Rapid turnover in plant sex chromosomes can provide opportunities to break this false impression and to explore the evolution of these turnovers. Recent results suggest that some plant lineages have dynamic sex-determination regions (SDRs) that show rapid turnover, resulting in poor conservation of the genetic mechanisms controlling sex [3,9,10]. For example, the SDRs of *Fragaria* octoploids provided the first case of translocation of a cassette of 14 kb of female-specific sequence among several chromosomes [11]. In *Silene*, section Otites has both female and male heterogamety and a possible change from female to male heterogamety within this section has been proposed [5].

Almost all species in the Salicaceae are dioecious, including those in *Salix* and *Populus* [12]. However, both female and male heterogamety are found in this family [13,14,15,16]. Chromosome 19 (Chr19) has been shown to be male heterogametic in several *Populus* studies [13,17,18,19]. Although sex determination has been consistently mapped to Chr19 in *Populus*, other chromosomes also show sex association peaks [13]. In contrast, sex determination consistently maps to a single major locus in *Salix*, where single association peaks have been identified on Chr15 [14,15,20] and Chr7 [21]. Multiple locations of sex-specific markers in *Populus* were proposed to be associated with the erroneous assembly of portions of the SDR in the reference genome [13]. Furthermore, the SDR in *P. trichocarpa* was inferred to be small and compact with less than 20 genes spanning ~100 kb on Chr19 [13], in contrast to the SDR of *S. purpurea*, which contains 488 genes and spans over nearly 7 Mb [20]. However, the previous results in *Populus* are based mainly on fragmentary de novo assembly based on short-read sequences and alignment of short-read sequences to a reference genome derived from a female individual, which would lack the SDR in this XY species [13,22]. More recently, assemblies of the SDRs of *P. deltoides* and *P. tremula* demonstrated that inverted repeats of fragments of a type-A response regulator are present in both species and that this fragment is likely responsible for silencing the full-length gene in males, thereby implicating this gene as a possible master regulator of sex in *Populus* [23].

In this study, we established a new assembly derived from a male *P. trichocarpa* clone. By identifying sex-linked genetic markers in this new assembly, we demarcated the sex-determination region on the Y chromosome and described the genomic composition of this Y-SDR in detail. We also inferred the age of the SDR from the substitution rates estimated from the terminal repeats of autonomous long terminal repeat (LTR) transposons. Finally, we tested if a shared sex-determining element is present in both *Populus* and *Salix*. With these findings, we provide evidence that the diverse sex-determining systems in *S. purpurea*, *P. tremula*, and *P. trichocarpa* are independently derived, despite sharing key features.

## 2. Methods

### 2.1. Initial Genome Assembly

Clone “Stettler-14” is a male *P. trichocarpa* tree growing near Mt. Hood, Oregon. The tree was originally collected as part of a study to determine the rates of somatic mutation and variation in methylation status [24]. The genome was sequenced to ~120× depth using PacBio technology, with an average read length of 10,477 bp. The genome was assembled using CANU v1.4 and polished using QUIVER [25]. The assembled genome contained 392.3 Mb of sequence and the contig N50 was 7.5 Mb. The genome assembly also contained ~232.2 Mb of alternative haplotypes. Full details of the assembly and annotation can be found in [24].

### 2.2. Variant Calling of Individuals from Natural Population

One hundred unrelated *P. trichocarpa* individuals of each sex were selected from a larger population that covers the range of the distribution [26]. The 2 × 100 bp resequencing reads of each individual were aligned to sequences in the main Nisqually-1 genome from the male reference genome Stettler-14 using Bwa mem 0.7.17 [27] with flags -M -t 8 -R. Duplicated reads were marked with MarkDuplicates from Picard [28]. The median depth of these alignments was approximately 11X per sample [26]. These alignments were used to retrieve variants through the HaplotypeCaller of GATK [29]. VariantFiltration of GATK was applied to filter variants with “AF < 0.01 || AF > 0.99 || QD < 10.0 || ExcessHet > 20.0 || FS > 10.0 || MQ < 58.0” in the -filter-expression flag. These settings cause a SNP to be removed if (1) allele frequency is lower than 0.01 or above 0.99, (2) the quality score normalized by allele depth is less than 10, (3) Phred-scaled *p*-value for an exact test of excess heterozygosity is greater than 20, (4) the phred-scaled *p*-value using a Fisher’s exact test to detect strand bias is over 10, or (5) RMS Mapping Quality is less than 58. The same steps were applied when the alignments were generated with reference sequences of alternative haplotypes from the male reference genome.

### 2.3. Sex-Association Analysis

All SNP variants generated from the previous steps were further selected with a minor allele frequency above 0.05 for sex-association analysis. The sex-association was performed with the same 100 females and 100 males using the Fisher’s exact test provided in plink v1.07 [30]. If the *p*-value of a tested marker was lower than the Bonferroni correction (with α = 0.05), then it was significantly sex-associated. Using the main genome assembly as a reference, 4,586,112 SNPs were tested, resulting in a Bonferroni correction threshold 1.09 × 10^−8^. The same steps were repeated to test 3,017,607 SNPs called from alternative haplotypes with a Bonferroni cutoff at 1.65 × 10^−8^.

### 2.4. Identifying the Sex-Specific Covered Region

To find hemizygous regions derived from sex chromosomes (either X- or Y-linked), we aligned the same reads from 100 unrelated individuals of each sex with Bwa mem 0.7.17 [27] to a reference that contain sequences from both the main genome and alternative haplotypes. Depth was calculated on the merged bam file from individuals of the same sex using Samtools-1.2 [31] and max depth was limited to 80,000. The median depth of 1-kb non-overlapping windows was calculated with an in-house python script. These 1-kb intervals were retained if the total median depth was no less than 400 to avoid inaccurate estimation of the depth ratio. If the depth ratio log_2_($\frac{F_{100}+1}{M_{100}+1}$) of the interval was smaller than −1, then the interval was considered as male-biased. If the log ratio was greater than 1, then it was considered as female-biased.

### 2.5. Genetic Linkage Mapping

Three half-sib families of male parents from a half-diallel designed cross (7 × 7) were used to generate three genetic maps [32]. A similar protocol as described above was used to call variants. For each half-sib cross, only markers in backcross configuration were used. Onemap v2.1.1 [33] was used to cluster markers into linkage groups and estimate the genetic distances. For computational reasons, markers of each cross were divided into two sets (even vs. odd indexes), so two maps were created for each cross, totaling six maps. In addition, a map generated from the interspecific cross 52,124 (*P. deltoides* × *P. trichocarpa*) with highly accurate Illumina Bead Array genotypes [34] was used to increase the accuracy. These seven maps were combined using ALLMAPS [35] to recreate the chromosomes.

### 2.6. Gene Annotation on the SDR and X-Linked Scaffold

To annotate potential coding genes that were missed by the automated annotation in the SDR and the X scaffold, the new Y-SDR contig and the X scaffold were submitted to the fgenesh [36] online service [37] with specific gene-finding parameters for *P. trichocarpa*. The predicted peptide sequences were searched against predicted proteins from *P. trichocarpa* v3.0 and *Arabidopsis thaliana* TAIR10 in Phytozome 12 [38] to find the closest homologous annotation. Only predicted genes that have at least one hit in either species were retained as valid predictions.

### 2.7. Estimation of the Divergence of the SDR

To identify allelic gene pairs for calculation of synonymous substitutions between the X and Y alleles, a reciprocal search of all annotated peptide sequences was performed by blastp with a limit of a maximum number of hits at 5, and MCScanX [39] was run with default parameters. Because of a lack of annotation of X haplotypes in Stettler-14, and to minimize the variation from different annotation software (see above for the methods used for annotating × haplotypes), we decided to use annotated genes from two X-haplotypes (a misplaced contig and scaffold_25) in the Nisqually-1 v4 genome, which was completed with the same annotation pipeline in Stettler-14. The synonymous and nonsynonymous substitution rates of each gene pair in each syntenic block (*d_S_* and *d_N_*, respectively) were estimated by aligning the sequences with CLUSTALW [40] using the yn00 function in PAML [41]. Tandemly duplicated genes with anomalously high *d_S_* values (>0.5) were removed from the analysis due to difficulties in determining orthology.

### 2.8. Identification of Recently Inserted LTR Retrotransposable Elements and Repetitive Elements

To identify recent insertions of transposable elements in the SDR and corresponding X interval, LTRharvest [42] was run with the sequence of the SDR (Y-SDR contig: 1–120,000 bp) and the X scaffold with the target site duplication restricted to 4 bp to 20 bp. To find the protein domains in the coding region, a protein domain search against Pfam-A domains (release 32) was performed using the hidden Markov model methods implemented in LTRdigest (–hmms flag) [43]. The same methods described in [20] were used to estimate the substitution rates between the LTR repeats. Briefly, the LTR repeats were aligned to one another, and the time since insertion was inferred using the number of substitutions.

Short tandem duplications were initially identified through TRF 4.09 [44] with 2 5 7 80 10 50 2000 -l 2 -d. Then, regions that contain no less than 1000 bp with a typical telomeric repeat motif (TTTAGGG)_n_-3′ or (CCCTAAA)_n_-3′ were designated as telomeric repeats [45]. A custom repeat library was derived using the RepeatModeler (v1.0.8) package, and repetitive elements were masked using RepeatMasker [46].

### 2.9. Expression of the Inverted Repeats

RNA-seq reads from flower tissues of three females (BESC423, 443, 842) and three males (GW9592, 9840, 9911) were retrieved from the poplar JGI gene atlas [47]. All sequence libraries used can be found in Supplementary Table S1. Each set of RNA-seq reads were aligned to the Stettler-14 reference genome with HISAT2 [48]. The alignments from the inverted repeats were visually checked for accuracy in the Integrative Genomics Viewer [49]. All replicates of the same stage of the same individual were merged with Samtools-1.2 [31]. The number of reads per site was retrieved with the depth flag by Samtools. Depth was calculated from the median of coverage in each 100 bp window for visualization.

### 2.10. Inference of Phylogenetic Relationship of the Homologous Sequences in the SDRs

Homologous genes in the Y-SDR of *P. trichocarpa* and the W-SDR in *S. purpurea* were identified using reciprocal Blastp searches using the predicted proteins from each interval. No genes had mutual best hits. We therefore performed Blastn with the nucleotide sequences from the SDR to identify shared nucleotide sequences. This revealed that most of the shared sequences are fragments of a cytokinin response regulator gene previously reported to be associated with sex in *Populus* [13,23]. Homologous sequences identified between the two SDRs were aligned by MUSCLE using default parameters provided in MEGA 5 [50], and the alignment was manually trimmed and adjusted to fix obvious alignment errors. The neighbor-joining method was used for building the phylogenetic tree with the substitution rate modeled by Kimura 2-parameter model provided in MEGA5, and the rate variation among sites was modeled with a γ distribution (shape parameter =1).

## 3. Results

### 3.1. Identification of Sex-Associated Scaffolds Based on SNP Associations

**Figure 1:**
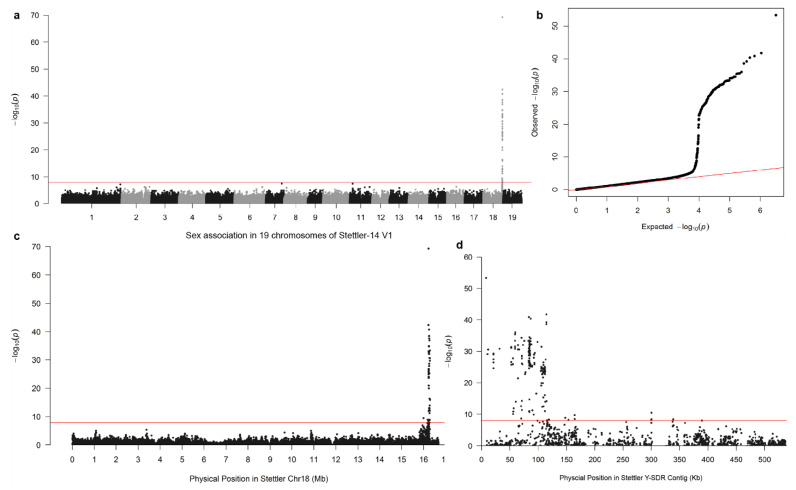
Sex association analysis with markers from the V1 main genome of Stettler-14. (**a**) Manhattan plot of *p*-values from sex-association analysis with 200 individuals in 19 chromosomes. (**b**) A quantile-quantile plot of the *p*-values the association analysis was displayed. For display, markers with no sex association were thinned to a minimum 100 bp spacing. (**c**) A close look at the sex-associated markers on chromosome 18. The red line indicates the Bonferroni cutoff (1.09 × 10^−8^) in (**a**–**c**). (**d**). A further zoom-in at the sex-associated markers on the Y-SDR contig. For convenience, plotted markers are a subset of the original dataset.

In the Stettler-14 V1 main genome, 4,586,112 SNP variants, called from GATK, were tested for association with sex by the Fisher’s exact test, yielding 119 sex-associated SNPs (*p*-value < 1.09 × 10^−8^), all of which were within a 300 kb stretch on Chr18 ranging from 15,993,536 bp to 16,289,766 bp in the V1 assembly (Figure 1a–c). Most sex-associated SNPs were found within the first 120 kb of one contig, hereafter referred to as the SDR. There were also some marginally significant sex-associated SNPs scattered around two regions at 160 kb and 300 kb (Figure 1d). In alternative haplotypes, 91 SNP variants (*p*-value < 1.66 × 10^−8^) were sex-associated from 3,017,607 tested SNP variants. These sex-associated SNPs in the alternative haplotypes highlighted scaffold_43 and scaffold_1208, with 33 and 56 sex-associated markers, respectively. Further alignment of scaffold_43 and scaffold_1208 also confirmed that they are alternative haplotypes of the SDR (Table 1). Scaffold_71 and scaffold_1121 were not considered to be sex-linked because there is only one sex-associated SNP in each of them.

### 3.2. Mapping of the Y-SDR to Chr19

**Figure 2:**
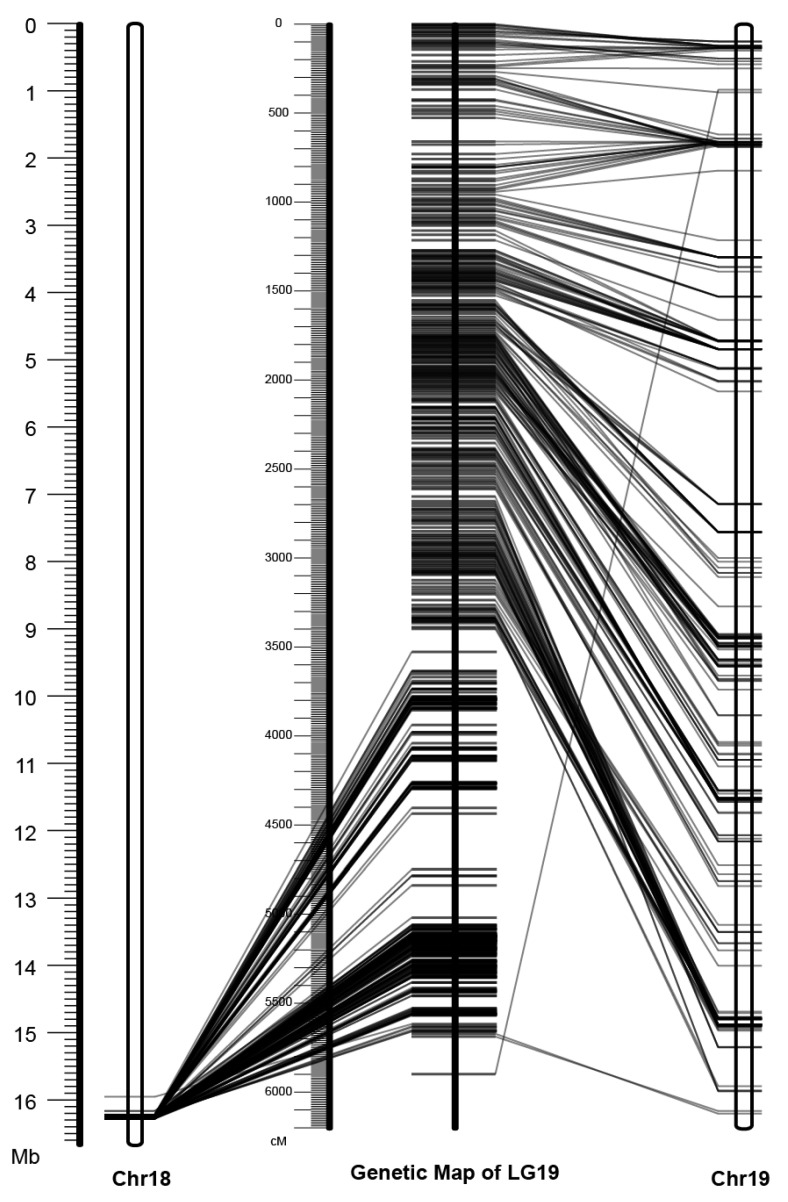
Comparison between the genetic map of chromosome 19 and the physical assemblies of chromosomes 18 and 19 in Stettler-14 V1. The physical assemblies of chromosomes 18 and 19 are on each side with unfilled rectangles, and the built genetic map of chromosome 19 is shown in the middle. Each horizontal tick represents a genetic marker and the corresponding physical position and genetic positions are connected with a line.

To evaluate the placement of this Y-SDR, we compared the order of markers in the consensus genetic map to the order in the physical assembly (Figure 2). The Chr18 placement was clearly incorrect based on this analysis, indicating that the contig containing the SDR should be placed on Chr19 (Figure 1), as was previously shown [13,22].

### 3.3. Confirmation of Male Heterogamety

**Figure 3:**
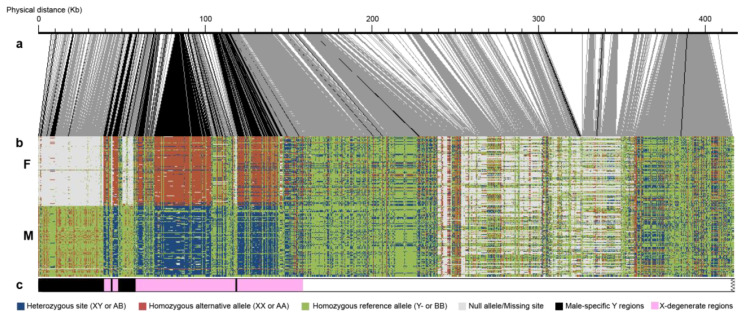
Genotype configurations of 200 individuals in the identified sex-linked region. (**a**) Schematic of the physical positions of SNPs. Black lines connecting to panel b indicate significant associations with sex. (**b**) The genotype configuration of the SDR and the adjacent pseuodoautosomal region. (**c**) Schematic interpretation of the genotype configurations in panel b.

The distribution of genotype configurations of the 200 sex-associated markers matches a male heterogametic (XX/XY) system (Figure 3). About 146 markers are configured as homozygous XX in females, while 138 markers are configured as heterozygous XY in males (Figure 3c). This confirmed the Y haplotype was present in this particular contig from the main reference genome, while alternative haplotypes were from the X chromosome. Additionally, the preponderance of female null alleles distributed from 10 kb to 50 kb showed the reference contained at least 40 kb of male-specific Y regions that are not covered in females (Figure 3b). Nearly all sex-associated markers occurred within 115 kb, suggesting that the SDR is confined to this region (Figure 3c).

### 3.4. Male-Specific Regions

To identify potential male-specific sequences in the assembly, we also performed depth analysis as described in [20]). In the main Stettler-14 genome, the depth analysis indicated that the same contig with sex-associated SNPs also contains 107 male-biased markers. The average of these 107 male-biased markers showed an extremely biased depth toward males with M:F depth about 9:1. This means that these markers are from a male-specific region with male coverage only. Further examination of the coordinates of these male-biased markers confirms that they are from the same contig where 119 sex-associated SNPs were found (Figure 3b). Among the analyzed alternative haplotype scaffolds, scaffold_43 and scaffold_1534 were found to contain 10 (out of 310) and five (out of 31) male-biased depth markers. However, for these male-biased markers, the depth of males is only about twice that of the females in both scaffolds, which follows an XX/XY system expectation. Since the reference used for depth analysis contains sequences from both the main genome and alternative haplotypes, we suspect that this could be an artifact due to the extra copy in the reference. Further alignment of scaffold_1534 confirms that this scaffold is an alternative haplotype of the Y-SDR contig with high sequence similarity (>99%) (Table 1).

### 3.5. Genomic Composition of the Y-SDR

**Figure 4:**
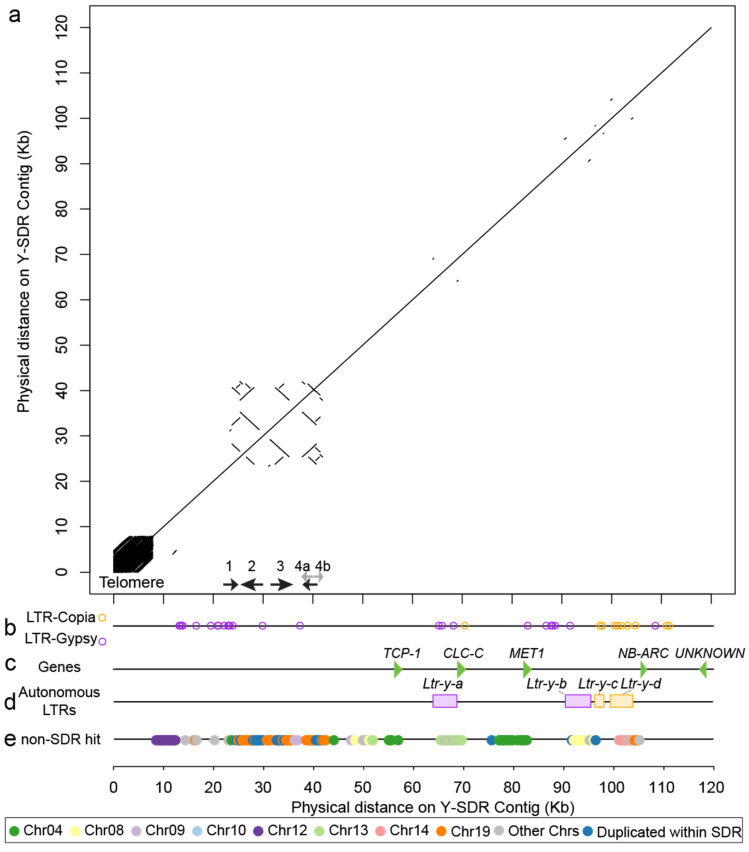
Dotplot and landscape of genomic contents of the Y-SDR in *P. trichocarpa*. (**a**) A dotplot of the alignment between the genomic sequence in the SDR in *P. trichocarpa* Stettler-14 to itself. (**b**) LTR-Copia and LTR-Gypsy elements identified from RepeatMasker were plotted as circles. (**c**) Five genes in the SDR are shown with green triangles. (**d**) Autonomous LTRs identified from LTRdigest/LTRharvest are shown with colored rectangles. Purple ones are from LTR-Gypsy superfamily and orange ones are from LTR-Copia superfamily. (**e**) Transposition identified in the SDR. Non-SDR hits of 200 bp sequence chunks from SDR. Only sequence chunks with single hit were kept. LTR: long terminal repeat.

Approximately 7800 bp at the end of the SDR was comprised of short tandem repeats of telomere repeat motif (TTTAGGG)n-3′ (Figure 4a). Similarly, one of its alternative haplotypes, scaffold_1208 contains about 4000 bp of tandemly duplicated telomeric repeats at one end. The Y-SDR is about 120 kb at the beginning of the chromosome 19 assembly, and it contains about 50 kb of sequence that is only present in male haplotypes (Figure 3b). The rest of the X-degenerate regions contain the majority of sex-associated markers identified above (Figure 3b). The male-specific regions consist primarily of fragments from Gypsy-LTR elements according to our analysis, while the X-degenerate region contains a mixture of Gypsy and Copia elements (Figure 4b). Additional identification of four autonomous LTRs allowed us to roughly estimate the minimal age of the SDR (Figure 4d). These Y-linked autonomous LTRs inserted into this region after the cessation of recombination. No autonomous LTR was found in the male-limited regions. All four LTRs are found to be inserted around the X-degenerate region but absent from X alternative haplotypes. Among these four autonomous LTRs, a Gypsy type LTR, *Ltr-y-a* shows the highest substitution rates of 33.95 substitutions per 1 kb. Using a mutation rate of 2.5 × 10^−9^ per year previously estimated from *P. tremula* [51], we estimated this oldest insertion occurred no later than 13.6 ± 3.7 SE million years ago. The remaining four LTRs have lower substitution rates (Table 2).

### 3.6. Inverted Repeats (IRs) in the Y-SDR

Five genes were annotated in the X-degenerate region of the SDR (Figure 4c, Table 3), including several sex candidates reported in a previous study of the SDR in *P. trichocarpa* [13]. The estimated synonymous substitutions rate (*d_S_*) between X and Y alleles differed among different genes. The gene Po14v11g055355m (function unknown) does not contain any synonymous substitutions but only nonsynonymous substitutions. Estimated *d_S_* values of the other three genes are 0.0176, 0.0224, and 0.0669, where *MET1* (Po14v11g055360m), which is furthest from the male-specific region, had the lowest substitution rate (Table 3). Interestingly, *TCP-1* (Po14v11g055363m) had the highest substitution rate, which is also the gene closest to the male-specific region. All of these *d_S_* values are substantially lower than the previous estimates of average *d_S_* of 0.146 ± 0.0022 SE between *S. purpurea* and *P. trichocarpa* [20]. A further search of the orthologous genes in a female reference (94006) of *S. purpurea*, by using these Y-SDR genes, showed that Po14v11g055355m was the only ortholog containing a hit on Chr19 in *S. purpurea*. The remaining genes do not have hits on Chr19 in *S. purpurea*. Both *MET1* and *TCP-1* have hits to Sapur.004G100800 and Sapur.004G101000 on Chr4 in *S. purpurea*. The best matches for these genes in *P. trichocarpa* are also on Chr4 (Figure 4e), which may indicate that they were transposed to Chr19 after divergence from *Salix*. The R-gene, Po14v11g055357m was excluded from the divergence analysis due to an excessive number of hits in the genome. When these genes were searched against a male *S. purpurea* reference, Po14v11g055355m and the *MET1* gene have hits to SpFC.19G000200 and SpFC.19G000100 from Chr19 in the male *S. purpurea* reference.

**Figure 5:**
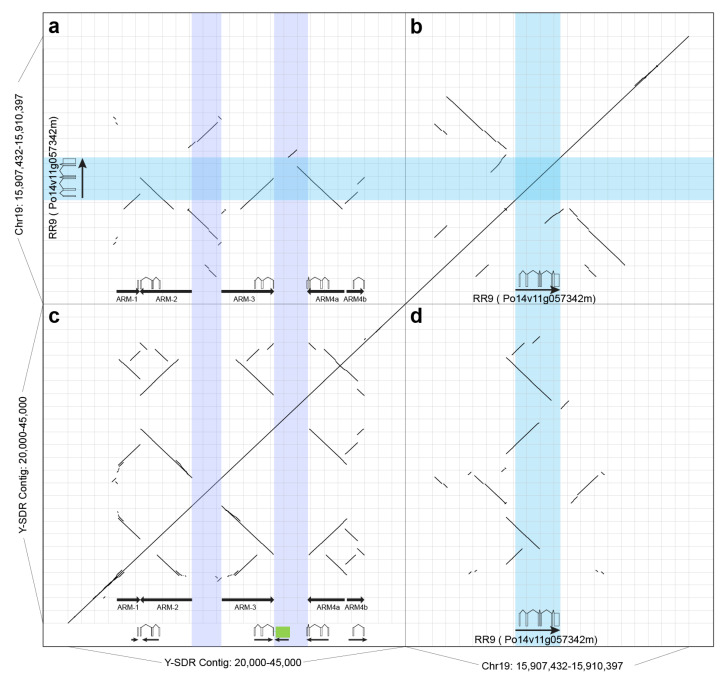
Dotplots of the male-specific inverted repeats in the SDR on the Y chromosome and comparison to the closest paralogous gene Po14v11g057342m. (**a**) A dotplot of the alignment between male-specific inverted repeats in the SDR in *P. trichocarpa* Stettler-14 to the region of *PtRR9* Po14v11g057342m on chromosome 19. (**b**) A dotplot of the self-alignment from the genomic sequence from 15,907,432 to 15,910,397 on chromosome 19 where *PtRR9* is located. (**c**) A dotplot of the self-alignment between the genomic sequence from 20 kb to 45 kb on chromosome 19 where male-specific inverted repeats are located. (**d**) A mirror image of the dotplot of a. Light purple colored bands show positions of two spacers in the SDR, and the cyan colored one shows the position of *PtRR9*. Schematics of the full-length *PtRR9* gene and repeated fragments are shown at the bottom. The green box represents a fragment of the *HEMA* gene.

In the Y-SDR, one of the features in the male-specific region is a cluster of five homologous arms arranged as inverted repeats (IRs) that might be derived from duplications and structural rearrangements (Figure 5a). By aligning the sequence from 20 kb to 45 kb of the Y chromosome, five arms were identified based on their sequence identity (Table 4 and Figure 5). The longest IR is formed between ARM-2 and ARM-3, and two arms have a similar length of about 3.8 kb with an identity of 93.3%. The two arms are separated by about 2 kb (Spacer-1), which is not homologous to these arms. ARM-4a and a partial sequence of the ARM-3 can also form an IR structure with a 2.7 kb spacer sequence (Spacer-2) (Table 4). ARM-1 and ARM-4b are shorter than the other arms but both contain homologous sequences of other arms (Figure 5).

All five arms have high sequence identity (>90%) to a gene located at the opposite end of Chr19 (*PtRR9*, *Po14v11g057342m*, or *Potri.019G133600* in *P. trichocarpa* V3). The closest homolog in *A. thaliana* is *ARR17* (AT3G56380), a type-A cytokinin response regulator. All five arms contain the first exon of this gene model, and none of them contain the full length of the gene model (Table 5). Both of the last two exons (exon 5 and exon 6) are absent from these arms. ARM-1 only contains the first exon, which does not contain any coding sequence (i.e., 5′-UTR). The only copy of exon 4 in the SDR is in the spacer between ARM-3 and ARM-4a with transcript-order along with exon 1–3 on ARM-4a (Figure 5c). All of the introns between exons in this region are also present in order based on the alignments to the gene model of *PtRR9*. The Spacer-2 between ARM-3 and ARM-4a also contains a fragment from Chr09 (Figure 4e and Figure 5c), which includes upstream sequence and the first exon of a Glutamyl-tRNA reductase gene (*Po14v11g032403m*, Chr09: 7,655,369–7,659,100), an ortholog of the *HEMA* (AT1G58290) gene in *Arabidopsis thaliana*.

**Figure 6:**
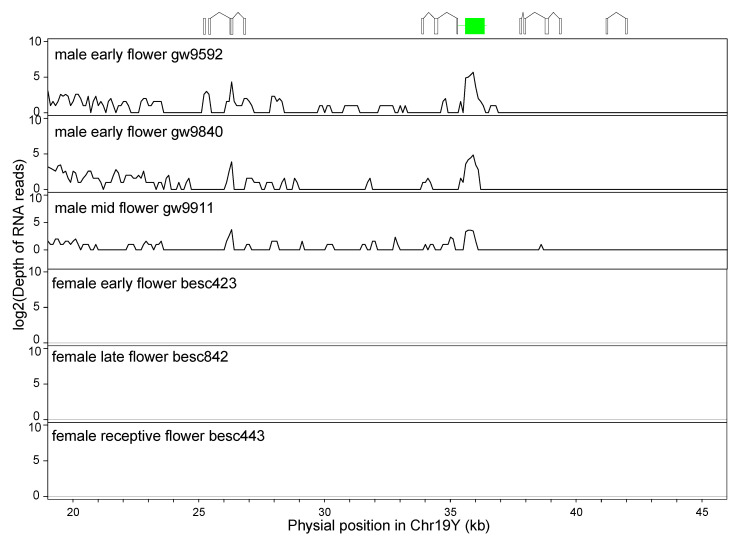
RNA-seq reads depth in the inverted repeats. The expression of fragments of the response regulator gene in the male-specific invert repeats was quantified by logarithmic of counts of RNA-seq reads in three male and three female individuals sampled from different flowering stages. Fragments of gene models are displayed above to help visualization, where the green box is a fragment of *HEMA*, and open boxes are exons duplicated from the *PtRR9* gene.

The expression of these IRs was detected by using RNA-seq of floral tissues from three males (Figure 6). We found male-specific expression in the region from 20 to 40 kb on Chr19. The fragments derived from the first exon of *Po14v11g032403m,* a homolog of *ATHEMA* in the Spacer-2 between ARM-3 and ARM-4a, showed expression in both the middle and late floral development stages. The fragments of exon1, exon2, and exon 3 from ARM-2 and ARM-3 were expressed in all three samples (Figure 6). Thus, these IRs are transcribed into RNA. However, based on alignment to the full-length mRNA, they are unlikely to code for a protein based on the presence of frameshift mutations and/or lack of a start codon.

**Figure 7:**
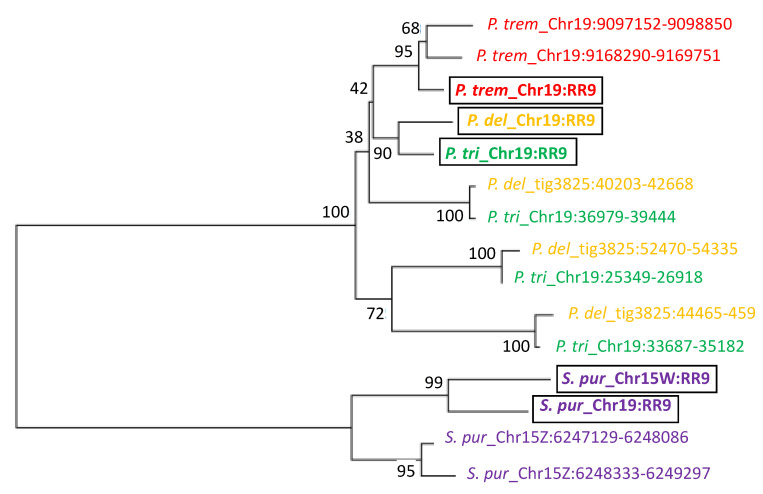
Phylogenetic relationship of homologous *PtRR9* fragments. A neighbor-joining tree was constructed based on sequence alignment of homologous portions of the *ARR17* gene (*RR9*) in *P. deltoides* (*P. del*), *P. tremula* (*P. trem*), *P. trichocarpa* (*P. tri*), and *S. purpurea* (*S. pur*). Species are color-coded. RR9 represents the full-length gene (bold, boxed). The genomic position is indicated after the underscore, where *Chr* represents a chromosomal scaffold, *tig* represents an unplaced contig, and positions of the inverted repeat fragments are indicated after the chromosome or contig identifier. Bootstrap values at the nodes were estimated with 1000 replicates in MEGA.

Given the presence of homologous response regulator genes or gene fragments in inverted repeats of the SDRs of *P. trichocarpa*, *P. deltoides*, *P. tremula*, and *S. purpurea*, we decided to test if these regions were derived from the SDR of a shared common ancestor or if they occurred independently in each lineage after species divergence. We constructed a phylogenetic tree using male and female autosomal copies and a representative sequence from the IR of each species (Figure 7). The *P. deltoides* and *P. trichocarpa* SDR repeats are syntenic and the individual repeats from the two species are in the same clade, suggesting a shared origin. In contrast, the *P. tremula* and *S. purpurea* inverted repeats are each in their own clades, together with the full-length genes, suggesting an independent evolutionary origin (Figure 7).

## 4. Discussion

Determining the ages and sizes of the SDR in non-model species is difficult, even with genome sequencing [52]. Here, we showed that the SDR in *P. trichocarpa* is quite small at approximately 115 kb, similar to the estimate of Geraldes et al. [13] based on short read assemblies. Our improved assembly coupled with estimation of depth of coverage across the genome shows that the male-specific region (i.e., regions that are present in Y but absent in X) is at least 40 kb, whereas Geraldes et al. [13] found four small male-specific contigs with an average length of 1877 bp. Such a small size of the SDR may simply reflect a recent origin of the SDR in *P. trichocarpa*: insufficient time has elapsed to allow for the expansion of this region [6]. This conclusion is supported by a general lack of homology between the SDRs in *P. trichocarpa* and *P. tremula*, suggesting that the loci evolved independent after divergence of the species [13].

In a previous analysis of sex association in *P. trichocarpa*, *POPTR_0009s08410* (*AtHEMA1*) on Chr09, and *POPTR_0019s15410* (*PtRR9*) on the other end of Chr19 were found in the regions significantly associated with sex [13]. The authors suspected the assembly of the genome could be erroneous, given the inconsistent locations of the association signals. In contrast with previous sex-linked signals over multiple chromosomes in the genome, the signals of sex-linked markers in our studies are well clustered within a 115 kb region. Using the complete assembly and annotation of the SDR region of Stettler-14, we showed that neither of these genes on Chr09 and the other end of Chr19 is associated with sex. Instead, transposed fragments of these genes are present in the SDR, thereby causing a false signal when a female is used as a reference genome (i.e., Y-SDR absent from the reference genome). This is a common problem for SDRs that contain sex-specific sequence, when the homogametic sex is used as a reference genome [20]. We have previously shown that the SDR of *S. purpurea* also contains abundant sequences transposed from autosomes [20]. Unfortunately, we could not identify reliable recent insertions of non-autonomous LTRs into the male-specific region in *P. trichocarpa* as we did for the female-specific region in *S. purpurea*, so we could not evaluate if these transpositions are related to LTR movements.

The Y-SDR in *P. trichocarpa* is different from the W-SDR in *S. purpurea* from several perspectives. The large size of the W-SDR was shown to be related to the accumulation of repetitive elements [20]. Also, the number of genes in the X-degenerate regions is different in the two species due to their dramatically different sizes. There are 156 Z-W homologous genes in the W-SDR of *S. purpurea*, but only 5 X-Y homologous genes in the *P. trichocarpa* Y-SDR. None of these genes were orthologous. By estimating the synonymous substitution rates of four gene pairs between the Y-SDR and the X-haplotype, we showed that the divergence after the arrest of recombination between X and Y haplotypes was likely to have begun after the split of *S. purpurea* and *P. trichocarpa* [20]. This again indicates that the age of the SDR might be young in both species, but further evidence from related species is needed to confirm this. Despite the differences between the Y-SDR and W-SDR, we discovered that a very similar sequence feature is present in the Y-SDR, which is the cluster of inverted repeats (IRs).

Indeed, the male-specific region is mostly composed of a cluster of homologous IRs that could be a result of transposition to the SDR followed by several duplications. This same arrangement was independently discovered by another group and was recently shown to be associated with sex in multiple *Populus* species [13]. Intriguingly, these IRs are homologous to a type-A cytokinin response regulator gene (*ARR17*) that is present in the SDRs of both genera. Previous genome-wide analysis of methylation showed that this response regulator gene was the only gene in the *P. balsamifera* genome that showed clear sex-specific methylation differences through its promoter and gene body [13]. This gene is also associated with sex in other *Populus* species [53,54]. Furthermore, silencing of this gene in *P. tremula* caused a female tree to produce male floral structures, suggesting that the *PtRR9* gene dominantly promotes female function [13]. This leads to a model in which the IR may encode a dsRNA species that is involved in RNA-directed DNA methylation (RdDM) [13,55]. Meanwhile, a methyltransferase gene (Potriv41g057386m) is present in the X-degenerate region of *P. trichocarpa*, but the role of this gene in *Populus* is not yet known.

The inverted repeats are clearly orthologous in *P. trichocarpa* and *P. deltoides*, which are sexually compatible. However, it is clear that despite sharing a similar IR structure, the evolutionary origin of the inverted repeats is independent in *P. tremula*, which is sexually incompatible with *P. trichocarpa* and *P. deltoides*. Strikingly, multiple inverted repeats of this gene also occur in *S. purpurea*, although in this case, they are located on Chr15 rather than Chr19 and are clearly of independent origin [20]. Within the SDR of *S. purpurea*, the full-length gene is repeated within large palindromes, and the four copies are nearly identical due to gene conversion (sequence identity above 99.5%). In contrast, the IRs found in the Y-SDR in *P. trichocarpa* show markedly lower sequence identity ranging from 90% to 95% between arms. The size of the homologous arms in *S. purpurea* is about 20 kb, with only a large (~7 kb) deletion on one of the arms [20]. In contrast, the size of the IR arms in *P. trichocarpa* is no more than 3.8 kb. These homologous IR arms also contain incomplete fragments from only one gene family, while homologous arms of the palindrome in *S. purpurea* contain four copies from five gene families and additional copies of other genes in the degenerated palindrome arms [20]. These differences indicate that the evolution and possibly the functions of the SDRs in the two genera might be different. These IRs in *Populus* are unlikely to play the same function as the ones from palindromes in *S. purpurea*. Interestingly, Chr15Z in *S. purpurea* also contains a truncated IR that is very similar in structure to those of *Populus*, but the function of this repeat in *Salix* remains unclear. In particular, it is unclear if this repeat causes silencing of the *SpRR9* gene in *Salix* males, as it does in *Populus*. The palindromic repeats on the *S. purpurea* Chr15W contain four full-length copies of the *SpRR9* gene, so these may be dominant to the effects of the *SpRR9* IR simply due to dosage. However, the W chromosome palindromes also contain a paralog of the Argonaute 4 gene, which is also part of the RdDM complex [55]. However, it is unclear what role this may play in establishing the dominant female-promoting function that is present in the ZW sex determination system in *Salix*. It is becoming increasingly clear that sex determination in the Salicaceae is mediated by epigenetic mechanisms targeting the cytokinin pathway. Additional functional data and analysis of the SDR from other Salicaceae species are required to test this hypothesis, and much remains to be determined about the ecological and evolutionary factors that drive this dynamic system.

## Figures and Tables

**Table 1:** Contigs containing alternative haplotypes of the sex-determination region (SDR).

seqID	Start	End	Ori	Size (bp)
**scaffold_1208**	1	85,028	-	85,028
**scaffold_1534**	95,029	169,954	-	74,926
**scaffold_43**	179,955	543,096	+	363,142

**Table 2:** Four autonomous long terminal repeat (LTR) identified in the Y-SDR.

LTR-ID	SuperFamily	SITE Count	Substitution Rate (SE)	Element Start	Element End	l/rLTR Length	TSD Motif	Pfam
*Ltr-y-a*	Gypsy	162	0.078(0.025)	64,125	69,169	175/162	aaat	Retrotrans_gag-223..315;RVP_2-479..564;RVT_1-724..861
*Ltr-y-b*	Gypsy	409	0.007(0.004)	90,484	95,727	409/409	tattt	Retrotrans_gag-262..351;RVP_2-512..601;RVT_1-746..906;rve-1255..1363;Chromo-1552..1599
*Ltr-y-c*	Copia	166	0.045(0.017)	96,610	98,445	166/166	tttc	UBN2_3-153..247;RVT_2-244..308
*Ltr-y-d*	Copia	294	0.065(0.016)	99,790	104,250	303/295	ttca	DUF4219-126..152;UBN2-204..281;gag_pre-integrs-514..572;rve-587..665;RVT_2-992..1139

Standard errors were obtained by a bootstrap procedure (1000 replicates). The Super family for each LTR retrotransposon was classified based on an online LTR classifier (http://ltrclassifier.ird.fr/LTRclassifier/form.html). TSD: target site duplication; LTR: long terminal repeat.

**Table 3:** Annotated genes in the SDR on the Y-linked contig in Stettler-14 with their homologous genes in other *P. trichocarpa* genomes.

GeneID	Start	End	Size	Strand	Description	Annotation in V3 Genome	Nisqually V4	dS(S.E.)	dN(S.E.)
Po14v11g055363m	52,354	56,656	4303	+	T-complex protein 1 subunit γ (TCP-1,CCT3, TRIC5)	Potri.018G138200; Potri.T046300	Potriv41g055126m;	0.0737(0.0136)	0.0016(0.0012)
							Potriv41g057391m	0.0600(0.012)	0.0008(0.0008)
Po14v11g055362m	59,327	69,212	9886	+	Chloride channel protein CLC-C	Potri.018G138100; Potri.T046200	Potriv41g055125m;	0.0117(0.0044)	0.0206(0.0078)
							Potriv41g057390m	0.033(0.0075)	0.0105(0.0025)
Po14v11g055360m	73,031	82,422	9392	+	similar to DNA (cytosine-5)-methyltransferase AthI (EC 2.1.1.37) (MET1)	Potri.018G138000; Potri.T046100	Potriv41g055122m;	0.0194(0.0041)	0.006(0.0013)
							Potriv41g057386m	0.0158(0.0036)	0.0057(0.0013)
Po14v11g055357m	96,006	105,907	9902	+	Archaeal ATPase (Arch_ATPase)//Leucine rich repeat (LRR_8)	Potri.018G137900	NA	NA	NA
Po14v11g055355m	116,621	118,021	1401	-	hypothetical protein	Potri.018G137700	Potriv41g055119m;	0(0)	0.0206(0.0078)
							Potriv41g057380m	0(0)	0.0236(0.0084)

**Table 4:** The physical positions of inverted repeats in Y-SDR contig.

Start (bp)	End (bp)	Size (bp)	ARMs
23,726	25,349	1624	ARM-1
25,381	29,199	3819	ARM-2
29,200	31,389	2190	Spacer-1
31,390	35,225	3836	ARM-3
35,226	37,885	2660	Spacer-2
37,886	40,646	2761	ARM-4a
40,531	41,958	1428	ARM-4b

**Table 5:** Fragments of response regulator genes in inverted arms compared to the complete paralogous gene Po14v11g057342m.

Po14v11g057342m (Chr19)	Size (bp)	ARM-1	ARM-2	ARM-3	ARM-4a	ARM-4b
exon1 (5′-UTR)	76	+	-	+	-	+
exon2	139	Absent	-	+	-	Absent
exon3	74	Absent	-	+(truncated)	-	+
exon4	78	Absent	Absent	Absent	-(Spacer)	Absent
exon5	71	Absent	Absent	Absent	Absent	Absent
exon6 (3′-UTR)	373	Absent	Absent	Absent	Absent	Absent

## Data Availability

The v1 assembly of *P. trichocarpa* clone Stettler 14 is available through Phytozome.

## Supplementary Materials

The following are available online at https://www.mdpi.com/2073-4425/11/8/843/s1. The library names of RNA seq reads used in this study can be found in Supplementary Table S1: List of RNAseq files and corresponding NCBI SRA accession numbers.
